# Role of Metallic Nanoparticles in Vaccinology: Implications for Infectious Disease Vaccine Development

**DOI:** 10.3389/fimmu.2017.00239

**Published:** 2017-03-08

**Authors:** Lázaro Moreira Marques Neto, André Kipnis, Ana Paula Junqueira-Kipnis

**Affiliations:** ^1^Department of Microbiology, Immunology, Pathology and Parasitology, Institute of Tropical Pathology and Public Health, Federal University of Goiás, Goiânia, Goiás, Brazil

**Keywords:** particulate vaccine, adjuvant, immune response, Th1, Th17

## Abstract

Subunit vaccines are safer but less immunogenic than live-attenuated vaccines or whole cell inactivated vaccines. Adjuvants are used to enhance and modulate antigen (Ag) immunogenicity, aiming to induce a protective and long-lasting immune response. Several molecules and formulations have been studied for their adjuvanticity, but only seven have been approved to formulate human vaccines. Metallic nanoparticles (MeNPs), particularly those containing gold and iron oxides, are widely used in medicine for diagnosis and therapy and have been used as carriers for drugs and vaccines. However, little is known about the immune response elicited by MeNPs or about their importance in the development of new vaccines. There is evidence that these particles display adjuvant characteristics, promoting cell recruitment, antigen-presenting cell activation, cytokine production, and inducing a humoral immune response. This review focuses on the characteristics of MeNPs that could facilitate the induction of a cellular immune response, particularly T-helper 1 and T-helper 17, and their potential functions as adjuvants for subunit vaccines.

## Introduction

Adjuvant selection for subunit vaccines is a key to increasing immunogenicity and, therefore, guiding stimulation of innate immunity and the development of the appropriate protective response to combat the microorganism of interest. Adjuvants are classified as particulate formulations, immunomodulatory molecules, or a combination of both characteristics. In addition to acting on the diversity of the humoral and cellular immune response, they can act in several different ways: by decreasing the vaccine dose, accelerating the immune response, or prolonging the immune response (1, 2). Among the seven approved vaccine adjuvants for human use, aluminum salts (alum), emulsions (e.g., MF59), and virosomes are particulate formulations. While alum induces efficient antibody (Ab) production and a predominant T-helper 2 (Th2) response, the other two have the capacity to induce T-helper 1 (Th1) and Th2 as well as Ab. Adjuvant system (AS) 01 and 04 used the combination of an immunomodulatory molecule and a particulate formulation composed of a Toll-like receptor 4 (TLR4) agonist, monophosphoryl lipid A that also induces Ab. The incorporation of alum in AS04 improved the humoral response, while the association of saponin (QS-21) and liposome in AS01 favored Th1 responses (3, 4). Imidazoquinolines (TLR7 and TLR8 agonists) and lipid A analogs (TLR4 agonists) are immunomodulatory molecules, capable of generating a Th1 response (5).

There is a demand for safe adjuvants capable of inducing efficient cellular immunity, especially Th1 and Th17, to be used against tuberculosis, leishmaniasis, malaria, and other diseases caused by intracellular microorganisms (1, 6). The majority of molecules with this type of adjuvanticity (Th1 driven) are related toward the response of danger receptors to trigger inflammation, thus safety and tolerance could be major barriers that prevent their use in human vaccines (7). However, comparing Alum and CpG/DNA adjuvants in human trials, only common adverse effects, including local site reaction, flu-like symptoms and headache were observed when CpG/DNA was used (8). Also, Verstraeten et al. (9), analyzing more than 30,000 individuals, who received vaccine-containing AS01, observed that only common side effects occurred.

Nanoparticles (NPs) are classically described as structures smaller than 100 nm and can be classified, based on their composition, as polymeric, inorganic, liposomes, immunostimulating complexes, virus-like particles, emulsions, or self-assembled proteins (10). They are made of different materials and differ in size, shape, and surface properties; interactions with biological systems, therefore, are varied, with several applications in modern medicine. In vaccinology, they are classically thought to have delivery and deposit properties. However, many NPs have been shown to stimulate immune responses, including cell recruitment, activation of antigen (Ag)-presenting cells (APCs), and induction of cytokine and chemokine release. The development of nanostructures and nanoadjuvants may therefore offer alternatives to currently used adjuvants once studies establish ways for them to elicit innate immune response and support the development of adaptive immune response in the context of vaccine formulations (10).

Metallic nanoparticles (MeNPs) are relatively non-biodegradable, have rigid structures, and possess simple synthesis methodology. Many have been studied for their immunological properties (11). However, there are still gaps in understanding the immune response generated by NPs, especially MeNPs. Few studies have compared NPs of different types and there is no standardization among published methodologies, which hampers comparisons of immunostimulatory characteristics. Several important characteristics, therefore, have not been well studied. For example, how chemical and physical properties (including material composition, size, shape, surface charge, and hydrophobicity) impact vaccine immune response (5). This review focuses on the use of MeNPs in formulations against infectious diseases, aiming to assess progress of their use in vaccinology and their possible applications as adjuvant.

## The Immune Response Generated by MeNP-Formulated Vaccines

Table 1 summarizes the articles that report the use of MeNPs as part of vaccine formulations against infectious diseases and the immune responses they elicited. A range of immune responses is required to fight a diverse group of microorganisms. The type of protective immune response can be simplistically divided based on the type of microorganism: extracellular bacteria and toxin, intracellular bacteria, viruses, fungi, and protozoa. Among the vaccines targeting extracellular bacteria and toxin, two were formulated with lipopolysaccharide (LPS) in glycopeptide Ag. The use of glycoantigen and LPS can trigger an intense response through TLR4 and B cell receptor activation; the presence of gold NPs (AuNPs) may have minimal influence on this response. However, in the work of Gregory et al. (12) and Torres et al. (13), the use of AuNPs in the formulation generated a different response, improving anti-LPS immunoglobulin G (IgG) response, decreasing bacterial burden, generating a more efficient humoral response, and improving animal survival, showing that AuNPs may influence immune response and protection.

**Table 1:** **Studies describing immune responses to vaccination with metallic nanoparticles, listed by NPs material and year of publication (*n* = 18 studies)**.

NP material	Complementary adjuvant	Animal model (route of vaccination)	Evaluation of immunogenicity	Reference
Gold		C57BL/6 (H-2b) and BALB/c (H-2d) mice used for protection experiments (intraperitoneal)	CD4+, IL-2+, and duration and avidity of total immunoglobulin G (IgG) (IgG1, IgG2a, IgG2b, and IgG2c)	Kaba et al. (14)
		BALB/c mice (intraperitoneal and subcutaneous)	IgG (total)	Chen et al. (15)
	Alum, CFA/IFA	BALB/c mice (subcutaneous)	IgG1, IgG2a, IgG2b, and IgG3	Parween et al. (16)
		Albino mice and rabbits (intraperitoneal)	IgG, circulant IFN-γ, and ROS *in vivo* generation by peritoneal macrophages	Staroverov et al. (17)
	Alhydrogel	BALB/c mice (intramuscular)	IgG1 and IgG2a titer, CD4 and CD8 activation, and IFN-γ release	Gregory et al. (12)
		C57BL/6 (H-2b) and BALB/c (H-2d) mice used for protection experiments (intramuscular/intraperitoneal)	Total IgG, IgM and IgA titer and avidity, and CD8+ memory population (effector, central, and long-term central)	Kaba et al. (18)
		C57BL/6 mice (intramuscular/intraperitoneal)	IgG1, IgG2c, IgG3, and IgE titers	Mccoy et al. (19)
		C3H/HeNJc1 mice (intraperitoneal)	IgG	Niikura et al. (20)
	CpG/DNA (TLR9 agonist)	BALB/c mice (intranasal)	IgG1 and IgG2a	Tao et al. (21)
	*Asparagus racemosus* extract	Swiss albino mice (oral)	Serum IgG, serum IgA, intestinal IgA, and fecal IgA	Barhate et al. (22)
	LPS (TLR4 agonist)	BALB/c mice (intranasal)	IgG1 and IgG2a	Gregory et al. (12)
	LPS (TRL4 agonist)	Rhesus macaques (subcutaneous)	IgG	Torres et al. (13)
	Advax™ adjuvant	BALB/c mice (intraperitoneal and intravenous)	T-helper 1, CD8+, and NK cells	Rodriguez-Del Rio et al. (23)
		BALB/c mice (subcutaneous)	IgG (total)	Dakterzada et al. (24)
Iron		SW mice (intraperitoneal, intramuscular, and subcutaneous), *Aotus lemurinus trivirgatus* monkeys (intramuscular)	Total Ab response, IFN-γ, and IL-4 (mice) and total Ab response (monkeys)	Pusic et al. (25)
Nickel		BALB/c mice (subcutaneous)	IgG response	Fischer et al. (26)
		BALB/c mice (subcutaneous)	IgG1 and IgG2a serum titer and IL-12/p40 and RANTES/CCL5 serum concentration	Wadhwa et al. (27)
		BALB/c mice (subcutaneous)	Specific serum IgG, IgG1 and IgG2a Ab titers and IFN-γ (splenocytes)	Yan et al. (28)

*Ab, antibody; Alum, aluminum salts; CFA, complete Freund adjuvant; IFA, incomplete Freund adjuvant; IFN, interferon; Ig, immunoglobulin; IL, interleukin; LPS, lipopolysaccharide; NK, natural killer; NP, nanoparticle; ROS, reactive oxygen species; SW, Swiss Webster mouse; Th, T-helper; TLR, Toll-like receptor*.

Using protein Ag, Barhate et al. (22) formulated a vaccine using AuNPs and toxoid Ag and demonstrated that their formulation could induce a mucosal and systemic IgG and IgA response. When co-administered with *Asparagus racemosus* extract, a botanically derived adjuvant, the response was further enhanced (22). Dakterzada et al. (24) developed a vaccine against *Pseudomonas aeruginosa* using the flagellin subunit and AuNPs that elicited an IgG response comparable to that induced by Freund Adjuvant. Flagellin is a TLR5 agonist but the recognition and signaling is structure dependent. This study, however, used only the 1–161aa from flagellin and its ability to activated TLR5 could not be maintained (24). Gregory et al. (12) used an F1 *Yersinia pestis* Ag conjugated to AuNPs that induced an Ab response with higher IgG2a associated with higher levels of interferon gamma (IFNγ), suggesting activation of Th1 cells.

Among the studies that used MeNPs in vaccine formulation, only one targeted intracellular bacteria (*Listeria monocytogenes*). The protective immune response against intracellular bacterial infections requires Th1 activation and, therefore, APCs activation and Ag presentation through major histocompatibility complex II (MHC II). To generate a Th1 response, an AuNP and Listeria Ag formulation were used in different strategies. Although the authors tested direct vaccination, when dendritic cells (DC), *in vitro* loaded with AuNP plus Listeria Ag, were adoptively transferred to a naïve animal, they induced Th1, CD8+, and natural killer (NK) cells that provided better protection against *L. monocytogenes* than the traditional vaccine approach (23).

In evaluating vaccines developed with MeNPs against viral infections, Niikura et al. (20) used West Nile virus (WNV); Tao et al. (21) used the extracellular portion of Matrix 2 protein (M2) of the influenza virus; Chen et al. (15) conjugated AuNPs with a 28 amino acid VP1-foot-and-mouth virus protein (pFMDV); and Staroverov et al. (17) co-administered AuNPs and partially purified enteropathogenic swine-transmissible gastroenteritis virus. All the above studies evaluated the Ab immune responses and all formulations demonstrated efficient humoral response induction. Tao et al. (21) also evaluated the addition of cytosine and guanine linked by phosphodiester unmethylated (CpG/DNA) and found that it improved Ab levels and animals’ survival rates. Another important feature of studies by Niikura et al. (20) and Chen et al. (15) was the use of various NP sizes and the demonstration that all different NP shapes were capable of inducing a humoral response. The levels of Ab were size dependent, but the results were inconsistent: the first study found that a 40 nm sphere was the most efficient Ab inducer and the second study found that the 8 nm and 12 nm spheres performed best.

A special case of the use of MeNPs was the use of nickel-functionalized nanolipoprotein particles (NiNLPs) by Yan et al. (28) and Wadhwa et al. (27) in combination with HIV Ag. NiNLPs are nanometer-sized nanolipoprotein particles with nickel incorporation into their surface in order to induce polyhistidine tagged proteins adsorption (29). They demonstrated that specific IgG (IgG1 and IgG2a) levels were greater than those obtained when alum was used in the formulation. Fischer et al. (26) used truncated WNV envelope protein Ag and found that a single dose vaccination induced a superior anti-WNV IgG response and improved protection against a WNV challenge (26). These responses were associated with nickel functionalization, described as a hapten, and triggered responses through activation of human TLR4 and intracellular transduction signals through myeloid differentiation primary response (MyD-88), nuclear factor-κB (NF-κB), and mitogen-activated protein kinase (MAPK), inducing pro-inflammatory responses [tumor necrosis factor (TNF)-α and interleukin (IL)-8] (30, 31).

For protozoan infections, Parween et al. (16), using *Plasmodium falciparum* merozoite surface protein subunit and AuNPs, evaluated the humoral immune response (IgG1, IgG2a, IgG2b, and IgG3) and found an intense IgG1 response compared with the alum formulation (16). Kaba et al. (14), using *P. berghei* circumsporozoite protein and AuNPs, generated long-lasting protective immunity with Th that produced IL-2 and mixed high avidity IgG1/IgG2a (Th2/Th1) (14). In other studies, these authors replaced Ag with *P. falciparum* circumsporozoite protein; vaccination was shown to induce protective cytotoxic (CD8+) cells, high avidity Ab titers, and specific effector memory, central memory, and long-term central memory CD8+ T cells in draining lymph nodes, spleen, and liver (18). This response was shown to be generated by DC cross-presentation, which had delayed fusion and interaction of endosomes with lysosomes caused by the AuNP formulation (19). Finally, PfMSP was used with dextran-coated iron oxide NPs (IONPs) and was capable of inducing a humoral response in two animal models (mouse and monkey). This response was also shown to inhibit parasite growth by 55–100% (25).

Most studies evaluated immunogenicity through measurement of the humoral immune response. According to their findings, the use of NPs was efficient in inducing an Ab-based response. Based on heavy chain structure, there are five types of Ab, each with a different role: IgG, IgM, IgA, IgD, and IgE. IgG and IgA can be subdivided as IgG1, IgG2, IgG3, IgG4, IgA1, and IgA2 based on additional small differences in their heavy chain. With regard to vaccination, humoral immunity is especially important in responding to infection by extracellular pathogens, toxins, protozoa, and viruses. Its importance is associated with the biological activities of immunoglobulins, including microorganism opsonization and phagocytosis; complement activation (32); toxins and microorganism neutralization (33); and mast cells and basophil activation (32, 34). In addition, immunoglobulins can help target cytotoxicity against infected cells (Ab-dependent cell cytotoxicity of CD8 T cells and NK). In some cases, however, the pathogens have the ability to evade the humoral system or can even use immunoglobulins as a way to facilitate cell invasion, as in the cases of *Mycobacterium tuberculosis* and *Leishmania* spp. (35, 36).

The studies described above clearly show that MeNPs (gold, iron, and nickel) can be used for vaccine development. Different MeNPs were used in conjunction with several Ag for distinct microorganisms and showed the ability to generate humoral and cytotoxic responses. Although the generation of IgG2a and IFN-γ shown in some studies are indicators of Th1 responses using MeNPs as adjuvant, further research is needed to specifically assess the role of different MeNP vaccines in Th1 induction.

## Important Physicochemical Characteristics of MeNPS as Activators of Immune Responses

**Figure 1:**
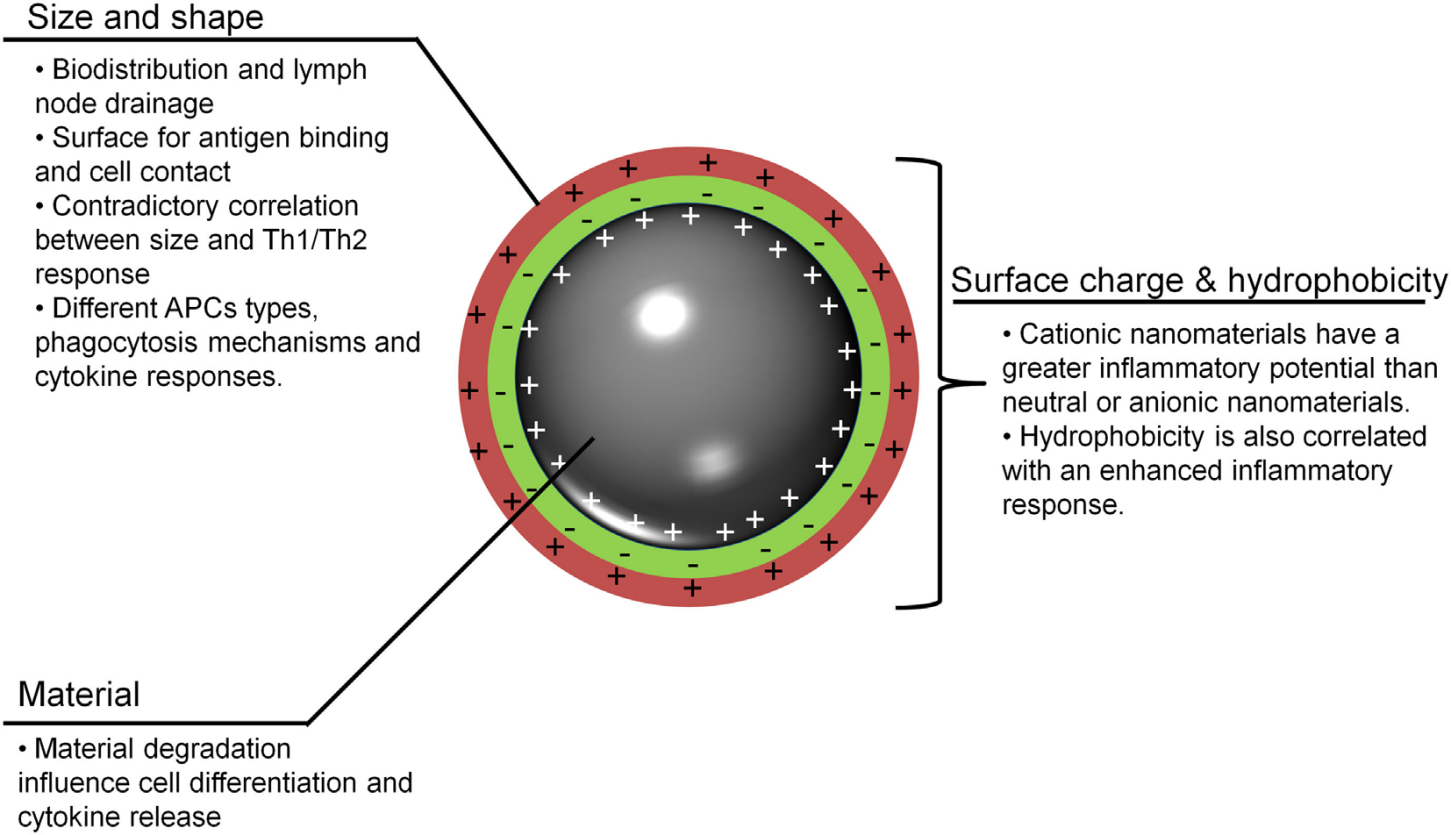
**Important nanoparticle characteristics for adjuvanticity**. To be recognized and to stimulate innate immunity, metallic nanoparticles (MeNPs) must have some physicochemical traits that allow for interactions with host cells and lead to the generation of a response. APCs, antigen-presenting cells; MeNPs, metallic nanoparticles; Th, T-helper cell.

To understand the possible uses of MeNPs as platforms for vaccines against infectious diseases, analysis is needed of the impact of different physicochemical characteristics of NPs on the innate immune response (Figure 1). Several strategies have included MeNPs as vaccine platforms, involving MeNPs of different materials (including gold, iron oxide, and nickel); shapes (including spheres, cubes, rods, and disks); sizes (from 2 nm to over 200 nm); and types of coating [e.g., citrate, chitosan, dextran, or cetyltrimethylammonium bromide/4-styrenesulfonic acid-co-maleic acid (CTAB/PSS-MA)].

The material from which an NP is made has a direct influence on the functions of APCs; gold NPs (AuNPs) have been most commonly used in vaccinology (Table 2). The most recent studies involving AuNPs demonstrate the effects of gold sodium thiomalate on macrophage function, showing lysosomal enzyme inhibition and reducing phagocytosis (37). Similar effects were seen in macrophages of several origins, which, when stimulated with AuNPs, showed diminished bactericidal activity against *Staphylococcus aureus* (38) and low or absent cytokine production IL-6, IL-10, and TNF-α (39, 40). Moreover, when splenocytes were stimulated with LPS, the addition of AuNP reduced IL-17 and TNF-α release (40). Some of these results raise the concern on the use of AuNPs as adjuvants, since these immunomodulatory properties can act inhibiting the generation of Th1. However, the response to AuNPs is also correlated with other physicochemical characteristics that will be discussed below, which may be tailored to improve immunostimulatory or immunomodulatory capacity.

**Table 2:** **Studies describing NPs and antigens used as vaccines against infectious diseases, listed by NPs material and year of publication (*n* = 18 studies)**.

NP material	Size in nm (shape)	Functionalization	Antigen (microorganism)	Reference
Gold	25 (sphere)		*P. berghei* circumsporozoite protein (*Plasmodium berghei*)	Kaba et al. (14)
	2, 5, 8, 12, 17, 27, 32, and 50 (sphere)	Citrate	pFMDV (foot-and-mouth virus)	Chen et al. (15)
	17 (sphere)	Citrate	PfMSP-1_19_ (*P. falciparum*)	Parween et al. (16)
	15 (sphere)	Citrate	Partially purified enteropathogenic STG coronavirus	Staroverov et al. (17)
	15.6 (sphere)	Citrate	F1-antigen (*Yersinia pestis*)	Gregory et al. (12)
	40 (sphere)		Pf CSP (*P. falciparum*)	Kaba et al. (18)
	35–40 (sphere)	Citrate	Pf CSP (*P. falciparum*)	Mccoy et al. (19)
	20 and 40 (sphere), 40 × 10 (rod), and 40 × 40 × 40 (cubic)	CTAB and PSS-MA	WNVE protein (WNV)	Niikura et al. (20)
	12 (sphere)	Citrate	Extracellular portion of M2 protein (influenza virus)	Tao et al. (21)
	40 (sphere)	Chitosan	Tetanus toxoid bulk from *Clostridium tetani*	Barhate et al. (22)
	15 (sphere)	Citrate	TetHC and modified LPS from *Clostridium tetani*	Gregory et al. (12)
	15 (sphere)	Citrate	LPS conjugated to FliC as glycoantigen (*Burkholderia thailandensis*)	Torres et al. (13)
	1.5 (sphere)		T cell epitopes, LLO_91–99_, and LLO_189–201_ (*Listeria monocytogenes*)	Rodriguez-Del Rio et al. (23)
	15 (sphere)	Citrate	Flagellin_1-161_ (*Pseudomonas aeruginosa*)	Dakterzada et al. (24)
Iron	20 (sphere)	Dextran	PfMSP-1_1-42_ (*P. falciparum*)	Pusic et al. (25)
Nickel	23 (discoidal)		Truncated WNVE protein (WNV)	Fischer et al. (26)
	199, 214, and 270 (capsule)		Gag p41 (HIV)	Wadhwa et al. (27)
	100 (capsule)		Gag p41 or p24/his-Nef (HIV)	Yan et al. (28)

*CTAB, cetyltrimethylammonium bromide; HIV, human immunodeficiency virus; LPS, lipopolysaccharide; NP, nanoparticle; Pf CSP, *P. falciparum* circumsporozoite protein; pFMDV:; PfMSP, *P. falciparum* merozoite surface protein; PSS-MA, poly(4-styrenesulfonic acid- comaleic acid); STG, swine-transmissible gastroenteritis; TetHC, Hc fragment (TetHc) of tetanus toxin; WNV, West Nile virus; WNVE, WNV envelope*.

Iron oxide nanoparticles have also been used as adjuvants. Iron is an important ion in the homeostasis of all cells and in generating immune responses to several microorganisms. The effect of IONPs phagocytosis have been explored in several studies, for example, M2 macrophages after exposure to IONPs induced reactive oxygen species (ROS), but after 24 h induced IL-10 production (41). The use of IONPs in BALB/c mice demonstrated the immunomodulatory capacity of this NP by diminishing splenocyte cytokine production (IL-4 and IFN-γ) (42) as well as suppressing the response to pancreatic Ag in diabetic mice (43). Sindrilaru et al. (44), however, showed that macrophages, under iron overloaded conditions, became unrestrained M1 (with an incomplete switch to M2 macrophages) and produced more TNF-α, which impaired wound healing and had an important role in the immunopathology of chronic venous leg ulcers. Consequently, IONP response seems to have direct correlation with time and dose, once iron overload seems to be a requisite to developed pro-inflammatory response and this aspect must be evaluated to avoid the inhibition of the desired immune response.

Other critical characteristics are the shape and size of NPs, which have a direct impact on vaccine efficiency, Ag load capacity, and interaction with cells (phagocytes and APCs). These characteristics have been studied in different NPs; Shah et al. (45) published a review focusing on the impact of size for alum, oil-in-water, emulsion, polymeric particles, and liposome adjuvanticity, but did not evaluated MeNPs. In the studies reviewed here, NP sizes range from 2 nm nanospheres to 270 nm nanocapsules. Two authors have evaluated the impact of size and shape for MeNPs (Table 2): Chen et al. (15) evaluated differences in immune response based on AuNP sizes (ranging from 2 to 50 nm nanospheres) and found that 8 and 12 nm were the most drained NP (15); Niikura et al. (20) went further and, using four different shapes of NP (20 nm sphere, 40 nm sphere, cube, and rod), showed that Ab responses and TNF-α were directly correlated with the specific NP surface area (the ratio of the total surface area per single NP volume). Furthermore, 40 nm spheres appear to be the most efficient in generating immune responses (IL-6 and IL-12) and granulocyte macrophage colony-stimulating factor production.

Surface charge and hydrophobicity are additional important NP characteristics for immune response induction and are directly influenced by NP functionalization (chemical modification of NPs surface by adding or replacing functional groups) and coating (Ag) (46). Most studies used citrate-coated NPs, but dextran and CTAB/PSS-MA have also been used; all three result in negatively charged (anionic) particles. Only one NP, revised here, used positive charged (cationic) functionalization [(22); Table 2]. The higher hydrophobicity of AuNP was shown to activate the innate immune system (TNF-α secretion) (47). Although the surface charge of other non-metallic NPs has been studied (48), to our knowledge the studies using MeNPs did not address the other characteristics associated with immune response induction. For non-metallic NPs, it appears that a positive charge signified a greater ability to induce immune responses than a negative charge. Interestingly, negatively charged non-metallic NPs were associated with Ag-specific tolerance (48). Further studies are needed to investigate whether or not the charge imputed by NP coating influences the immune response. Though the size and shape of MeNPs had little to no impact on the innate response elicited, coating modifications may improve the capacity of these molecules to influence immune responses. Finally, it is important to note that the majority of adjuvant characteristics were evaluated using non-metallic NPs.

## NPs as Adjuvants to Generate Th1 and Th17 Responses

T-helper 1 cells are associated with immunity against intracellular pathogens and the secretion of IFN-γ, which, in turn, is essential for the activation of mononuclear phagocytes, including macrophages, resulting in enhanced phagocytic activity (49). Th17 cells (IL-17A and IL-17F producer cells) are associated mainly with stimulation and chemotaxis of neutrophils to the site of inflammation. However, their function goes beyond this and includes the targeting of various cells types, including non-lymphoid cells and the stimulation of cytokine, chemokine, and prostaglandin production. Another characteristic of these cells is their memory effector subset, which is maintained in mucosal tissues for extended periods. This subset has high plasticity and is able to transform into Th1 or Th2 phenotypes depending on the cytokine milieu at mucosal sites. This diversity of function and actuation make Th17 cells very important in defense against several microorganisms, mainly those acquired through mucosal routes (49, 50).

T-helper 1 and Th17 cells have their own distinct sets of functions and differentiation factors. Both cell types require T cell receptor downstream activation by Ag presentation cells through MHC II and co-stimulatory molecules (6). Consequently, cytokine release during Ag presentation is correlated with the type of adaptive immune response generated. While Th1 differentiation requires stimulation by IL-12, Th17 generation requires transforming growth factor-β and IL-6. However, this generation is influenced by other factors and how MeNP are involved in the possible induction of Th1 or Th17 will be discussed below.

In this review, only one study investigated the development of the direct Th1 (type 1 T helper cell) and Th17 response. Using a Listeria Ag, Rodriguez-Del Rio et al. (23) showed that in contrast to Advax™ adjuvant alone, a combination of 25 nm AuNPs and Advax™ was capable of inducing the highest Th1 response. Pusic et al. (25) immunized mice with IONPs covered with rMSP1, a *P. falciparum* merozoite Ag, and showed that after immunization (intramuscular, subcutaneous, or intraperitoneal), production of IL-4 was greater than that of IFN-γ, suggesting a predominant Th2 response (although the cellular immune response was not directly evaluated).

The first major determinant in generating Th1 and Th17 populations is the route of vaccine administration, which dictates the cell dynamic and initial response to the vaccine. For example, Mohanan et al. (51), in a cross-sectional study using a liposome plus Ag (OVA) vaccine formulation, compared intradermal (high IgG1; intermediate IgG2; and IFN-γ), intralymphatic (high IgG1, IgG2, and IFN-γ), intramuscular (high IgG1; intermediate IgG2 and IFN-γ), and subcutaneous (high IgG1; low IgG2 and IFN-γ) routes of administration (51). The predominant Th1 response to administration through the intradermal route was most likely due to the cooperation between Langerhans cells, the primary innate immune response cells and keratinocytes that may also be stimulated by the formulation. These elicited the production of cytokines and chemokines that helped in the activation of other APCs (52).

The early phase of vaccination is characterized by recruitment of neutrophils and monocytes to the site of inoculation. Both cell types can also act as APCs, delivering Ag-specific and co-stimulatory signals to T cells. Their collaborative endeavors have been found to modulate (positively or negatively) the activity of different effector T cell subsets (53, 54). Neutrophils are the first cell lineage to migrate to inflammation sites and, when stimulated, they produce cytokines and chemokines that will attract and activate other cell types. For example, neutrophils were shown to be an important inducer of Th1 and Th17 cells (55), but their role in cytokine secretion is much broader (56). Moreover, signals may elicit different function in neutrophils and therefore, influence the quality of T cell responses. For example, AuNPs have been described as capable of inducing neutrophil extracellular traps, which act as damage-associated molecular patterns and stimulate immune system through DNA receptors such as TLR9 (57). Upon stimulation by NPs (TiO2—titanium dioxide—and alum), Duffin et al. (58) demonstrated neutrophil influx to the lungs and also induced production of IL-18. Silver NPs were also shown to be capable of interacting with neutrophils, inducing apoptosis of these cells, and inducing caspase-1\caspase-4 partially dependent IL-1β secretion (59). In another study, cobalt and nickel NPs were shown to induce higher nitric oxide, TNF-α, and CXCL2 chemokine production, by human peripheral blood neutrophils, than titanium NPs (TiO2NP) (60). Nonetheless, TiO2NPs also induced polymorphonuclear cell activation through phosphorylation of several proteins, including p38 MAPK and extracellular signal-regulated kinases-1/2 (Erk-1/2), which were associated with increased neutrophil life-span and production of several cytokines and chemokines (61).

Classically, APCs, macrophages, and DCs act at the site of vaccine inoculation by sensing foreign agents, through TLRs and other receptors, and triggering inflammation. APCs play a key role in the initiation, maintenance, and selectivity of inflammation, through their three major functions: endocytosis, Ag presentation, and production of various cytokines and growth factors (1). The main family of pattern recognition receptors in microbial recognition is the TLRs, part of the family of transmembrane proteins, which affect the transcription of genes involved in inflammatory and immune response-enhancing cellular activities such as phagocytosis, endocytosis, cytotoxic functions, and cytokine production (62, 63).

The adjuvants most frequently used for the induction of Th1 and Th17 responses are TLR agonists, such as AS04, CPG/DNA, and others. MeNPs seems to have capacity to induce the expression of Toll-like receptors, such as TiO2NPs and zirconium oxide NPs that have been described to enhance TLR3, TLR7, and TLR10 expression in macrophages (64) and TLR2 and TLR4 in mouse liver cells (65). Zinc oxide NPs (plus OVA) generated an inflammatory response in BALB/c mice and also improve TLR-2, -4 and -6 expression, followed by activation of Src family kinases (66). Consequently, TiO2NPs and IONPs were shown to induce DC upregulation of co-stimulatory molecules (MHC II, CD80) (25, 67, 68), which can also be related to TLR stimuli pathways. However, none of these works demonstrate the direct interaction of NPs with TLR (using Knock-out mice, agonists, or antagonist molecules) thus, this interaction must be further studied.

The next step in the generation of adaptive responses is the tailoring of cytokine secretion by APCs at immunological synapses, which will guide the development of the response. Several NPs have been reported to trigger cytokine and chemokine production, which may be used as biomarkers for immunotoxicity (69). Among those described, TiO2NPs were used in mimetic systems composed of blood vein endothelial component (including PBMC) and was reported to trigger pro-inflammatory cytokines (IL-6, IFN-γ, and TNFα) (67); Zinc oxide NPs were shown to be preferentially associated with monocytes and, when used in PBMC, induced IFN-γ, TNF-α, and IL-12 cytokine production (70); AuNP-stimulated bone marrow-derived DC produced IL-6, TNF-α, and IFN-γ (20); and IONPs were shown to induce the activation of APCs with an increase of IL-6, TNF-α, IFN-γ, and IL-12, as well as chemokines. The response generated by IONPs, however, was weaker than that generated by the positive control LPS which may be beneficial in controlling possible side effects (25).

The generation of a cellular response associated with protection against intracellular pathogens is the ultimate goal of vaccination. However, the direct effects of NPs on cellular responses have been evaluated in only a few studies. TiO2NPs were shown to activate and induce proliferation of naïve CD4+ T cells and to generate a pronounced Th1 response with IFN-γ and TNF-α production, associated with pro-inflammatory cytokine production (IL-6, IL-1a, IL-1b) and DC maturation (CD86+ and CD83+ expressions increase). Schanen et al. (71) hypothesized that the oxidative capacity of an NP could impact the response and trigger pro-inflammatory (oxidant capacity) or anti-inflammatory (antioxidant capacity) responses. This oxidant effect could control ROS generation and thus control downstream pro-inflammatory effects while antioxidants prevent the initiation of the innate immunity in LPS-stimulated macrophages (71). This study was, however, conducted with mitogens (non-specific stimuli) and not with vaccine stimuli, but nevertheless serves as a warning about the direct action of NPs, not only on the innate immune system but specifically on T cells.

## Conclusion

**Figure 2:**
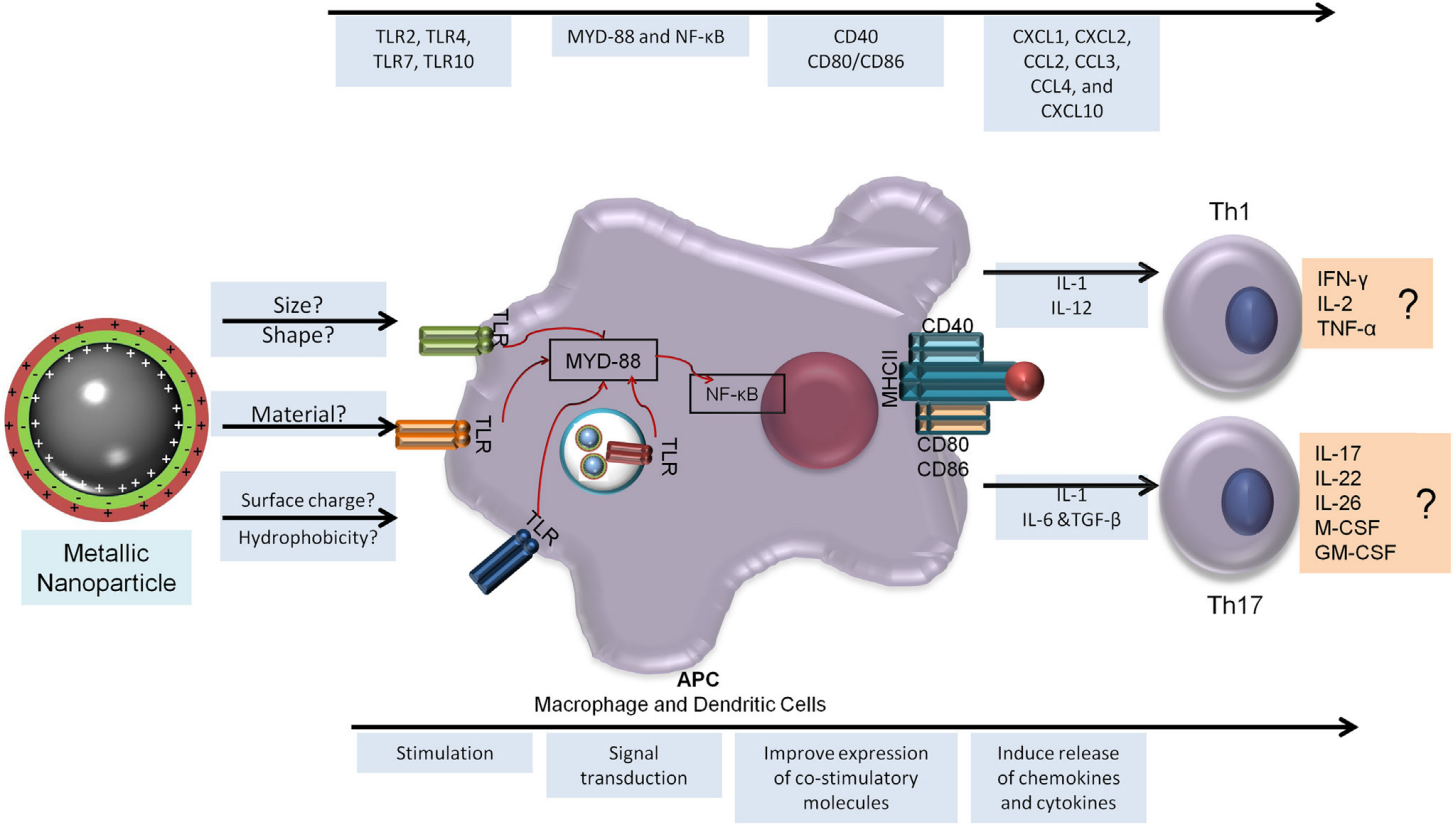
**Metallic nanoparticles adjuvanticity and its prediction capacity to generate T-helper 1 (Th1) and Th17 responses**. To generate a cellular immune response, the NP must be able to be recognized by the host innate immune response and stimulate a sequence of events that will lead to the release of a specific milieu of cytokines and better antigen presentation (bottom arrow). In the top arrow is the immune response elicited by metallic nanoparticles to aid Th1 and Th17 generation. NF-κB, nuclear factor kappa B; CCL, chemokine ligand; CXCL, chemokine (C-X-C motif) ligand; GM-CSF, granulocyte macrophage colony-stimulating factor; IFN, interferon; IL, interleukin; M-CSF, macrophage colony-stimulating factor; MYD, myeloid differentiation factor; TCR, T cell receptor; Th, T-helper cell; TLR, Toll-like receptor; TNF, tumor necrosis factor.

There is enough evidence to suggest that MeNPs are not only particulate formulations but also immunostimulatory molecules with several studies demonstrating their capacity to generate humoral and cytotoxic responses. MeNPs clearly have immunostimulatory capacity and can induce several reactions in all phases of vaccine development. These capabilities correlated with NP physicochemical characteristics such as size, charge, and hydrophobicity, but there are several gaps in our understanding of their mechanism of actions and how they may lead to adjuvanticity, immunomodulation, or tolerance to the Ag formulated with NPs. There are also evidence of MeNP being capable of help in the generation of Th1 and Th17; Figure 2 presents an overview of the generation of these cells subsets and the possible role of MeNP in this induction.

## Author Contributions

LN designed the review and wrote the first draft. AJ-K edited the first draft and critically reviewed the manuscript. AK edited the first draft and critically reviewed the manuscript. All authors read and approved the final version of the manuscript and agreed to submission.

## Conflict of Interest Statement

The authors declare that the research was conducted in the absence of any commercial or financial relationships that could be construed as a potential conflict of interest.
